# The Pathologic Actions of Phosphate in CKD

**DOI:** 10.34067/KID.0000000820

**Published:** 2025-04-17

**Authors:** Abul Fajol, Christian Faul

**Affiliations:** Section of Mineral Metabolism, Division of Nephrology, Department of Medicine, Heersink School of Medicine, University of Alabama at Birmingham, Birmingham, Alabama

**Keywords:** CKD, hyperphosphatemia, vascular calcification

## Abstract

CKD is associated with high serum levels of phosphate (also called hyperphosphatemia), which is a main driver of soft tissue calcifications and potentially other pathologic changes that are associated with CKD. However, it remains unclear in what form and through which mechanisms and targets elevated phosphate can damage cells and tissues. Rises in serum phosphate levels are accompanied by changes in the endocrine regulators of phosphate metabolism and result in the formation of calcium-phosphate crystals, and all three events can have pathologic actions on various tissues. Furthermore, tissues can accumulate phosphate from the circulation, and cells can generate free phosphate in their environment independently from circulating phosphate, which both result in local elevations of phosphate that could also contribute to tissue damage. It is important to better understand the various scenarios underlying the pathologic actions of hyperphosphatemia, as some of them suggest that measuring extracellular serum phosphate, which is the gold standard to estimate overall phosphate status of the body, is not sufficient to do so. Understanding the pathologic actions of phosphate on a conceptual level should not only help to design more efficient detection tools for phosphate but also to identify phosphate-induced pathomechanisms which could provide us with novel drug targets to tackle phosphate-driven pathologies in CKD. Here, we discuss the different concepts and scenarios that could underlie the widespread pathologic actions of hyperphosphatemia in CKD.

## Introduction

Inorganic phosphate is an essential mineral that is taken up through the diet and required by every cell type in the body to support over 1000 chemical reactions, such as the synthesis of DNA/RNA and phospholipids, the generation of the energy currencies ATP and phosphocreatine, and phosphorylation-mediated signaling events. About 15% of total body phosphate is present within cells, and they are mainly in mitochondria and in the endoplasmic reticulum.^1^ More than 80% of overall phosphate is stored in the extracellular matrix of bone in form of the calcium salt, hydroxyapatite, where it forms an important structural component of the skeleton. Phosphate homeostasis is controlled by the communication between the intestine, bone, kidney, and parathyroid gland.^2^ Phosphate uptake in the small intestine and phosphate excretion through the kidney are mediated by specific sodium-dependent phosphate (NaPi) transporters.^3^ Phosphate metabolism is mainly regulated by three endocrine factors, *i.e*., fibroblast growth factor 23 (FGF23), parathyroid hormone (PTH), and vitamin D, which are interconnected by complex feedback mechanisms.^4^ PTH and FGF23 target proximal tubular epithelial cells through PTH receptors and fibroblast growth factor receptor/klotho coreceptor complexes, respectively, thereby reducing renal phosphate reabsorption through NaPi-2a and NaPi-2c and lowering serum phosphate levels. By contrast, vitamin D increases phosphate uptake in the gut by upregulating NaPi-2b in enterocytes resulting in increased serum phosphate concentrations. Controlled phosphate homeostasis is necessary to ensure that all cells in the body are provided with phosphate through the circulation, while significant elevations of serum phosphate concentrations are avoided, since high phosphate has various pathologic effects.^5,6^

Since renal excretion is the only way for the body to release phosphate, impaired kidney function is associated with significant disturbances in phosphate homeostasis, and the development of high systemic phosphate levels (also called hyperphosphatemia), secondary hyperparathyroidism, and vitamin D deficiency are hallmarks of CKD.^2,7^ Reductions in kidney function also cause the development of hyperphosphatemia during the normal aging process.^8^ Furthermore, hyperphosphatemic states can result from rare genetic disorders, such as familial tumoral calcinosis. Hyperphosphatemia can also occur when intracellular phosphate is released rapidly because of tissue damage, as the case in rhabdomyolysis, hemolysis, and tumor lysis syndrome.^8^ High dietary phosphate intake by healthy individuals can significantly increase serum phosphate levels for several hours,^9,10^ showing that hyperphosphatemia can also occur in the absence of disease. Since the consumption of foods and drinks rich in phosphate-based additives is expanding with the Westernized diets, excess dietary phosphate intake and thereby potentially elevations in systemic phosphate levels in the general population are on the rise.^11^ In this article, we focus on CKD, which, among the mentioned pathologies and diseases, is the most studied, and potentially most frequent scenario of hyperphosphatemia. In CKD, hyperphosphatemia is a core parameter that is associated with all-cause mortality^2^ and a major risk factor for cardiovascular events, resulting in calcium-phosphate supersaturation and vascular calcifications.^12^ Because it is common in patients with CKD and clinically well described,^13^ vascular calcification is the best understood pathologic event caused by high phosphate levels.^14^

## The Pathologic Actions of Phosphate on a Cellular Level

Vascular calcification is an active cell-mediated process that is based on the activation of osteogenic gene programs in vascular smooth muscle cell (VSMC) in the vascular wall and their transdifferentiation into osteoblast-like cells.^14^ These changes include alterations in the extracellular matrix, such as collagen composition, which provide the turf for the calcification event. Calcifying VSMCs also release extracellular vesicles (EVs) containing calcium and phosphate which provide the substrate for calcifications. It is believed that intra-EV levels of calcium and phosphate increase until crystals form and burst the cell membrane, which then serves as the driver of hydroxyapatite formation in the extracellular matrix.^14^ It is well established that high levels of inorganic phosphate directly induce osteogenic differentiation of VSMCs and thereby serve as the initial trigger for vascular calcification.^15^ Furthermore, inorganic phosphate can induce a proinflammatory response in the vasculature, and together, the calcifying VSMCs and the inflammatory environment seem to synergistically drive the calcification process.^16,17^

Studies in various cell types other than VSMCs have linked elevations in extracellular phosphate concentrations to changes in gene expression and in cellular functions.^5^ This includes the phosphate-induced release of inflammatory cytokines from hepatocytes^18^ and the release of osteopontin and FGF23 from osteoblasts and osteocytes.^19,20^ Combined, these studies suggest that inorganic phosphate can modify gene expression and functions in cells and at high concentrations potentially act as a pathogen that directly induces cell damage. However, the precise mechanisms underlying the induction of these phosphate-induced cellular events remain unclear. It is unknown if elevations in circulating phosphate levels result in increased uptake of phosphate by cells, thereby causing elevations in intracellular phosphate levels, which could alter gene expression and have negative effects on cell function.^21^ It is also possible that elevated phosphate acts as an extracellular signal mediator that induces signal transduction events across the cell membrane without entering cells, thereby causing changes in gene expression and cell function.^5,22^

**Figure 1:**
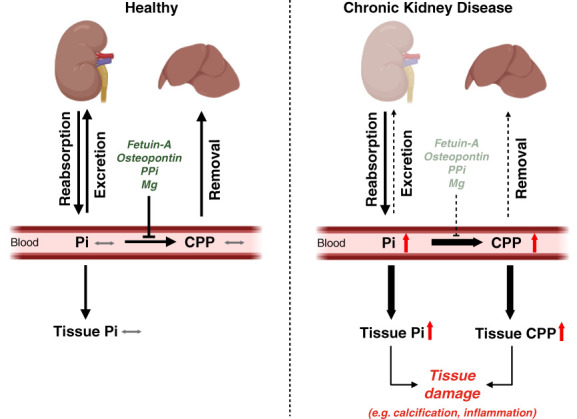
**The regulation and effects of Pi and CPPs in CKD.** In heathy conditions, renal excretion and reabsorption of inorganic Pi maintain blood Pi at normal levels. Thereby, tissues receive sufficient Pi to maintain cellular functions and structures, while tissue Pi elevations with potential cytotoxic effects are avoided. Fetuin-A, osteopontin, PPi, and Mg prevent the formation of calcium-Pi crystals in the blood and in soft tissues and thereby protect from ectopic calcifications in scenarios of systemic Pi elevations, for example after food intake. Fetuin-A binds calcium and Pi to form CPP, which are quickly removed from the circulation by the liver and are not deposited in tissues. In CKD, reduced renal excretion causes an elevation in inorganic Pi levels in the blood, which might result in increased Pi levels in tissues. CKD is also accompanied by reduced levels of fetuin-A, osteopontin, PPi, and Mg, which together with high Pi levels and reduced hepatic clearance capacity might cause the appearance of CPPs in the blood and eventually in tissues. The accumulation of inorganic Pi and CPPs in tissues and cells induces various pathologic changes, such as local calcification and inflammation. CPP, calciprotein particle; Mg, magnesium; Pi, phosphate; PPi, pyrophosphate.

The ability of soluble phosphate to form nanocrystals with calcium resulting in the generation of solid particles might underlie some of the effects of phosphate elevations that have been observed in experimental studies and that might drive some of the pathologic actions of hyperphosphatemia in patients with CKD.^23–25^ When concentrations of calcium and phosphate exceed solubility limit, insoluble calcium-phosphate crystals are generated instantaneously, which can grow and eventually precipitate as hydroxyapatite.^26^ This process is a chemical reaction that can occur in the absence of cells. However, calcium-phosphate crystals cannot grow in blood because of the presence of fetuin-A, which is a liver-derived protein that acts as a mineral binding protein and calcification inhibitor. Fetuin-A absorbs calcium-phosphate crystals and thereby prevents their growth and maturation.^27,28^ Calcium-phosphate and fetuin-A form calciprotein particles (CPPs) which are colloid particles that are dispersed in solution^24^ and that are cleared rapidly from the circulation by the liver and spleen^29–31^ (Figure 1). In the liver, Kupffer cells and hepatocytes seem to take up CPPs and mediate CPP clearance.^31^ Since uncontrolled formation of calcium-phosphate crystals outside of bone tissue would be detrimental, the body has generated several mechanisms to protect from ectopic calcifications. This includes not only fetuin-A but also osteopontin, which is produced by many tissues and, like fetuin-A, acts as a phosphate binding protein and buffer.^32^ Furthermore, pyrophosphate (PPi) and magnesium block the chemical reaction underlying the formation of calcium-phosphate crystals.^33,34^ In CKD, systemic levels of fetuin-A, PPi, and magnesium are reduced,^33,35,36^ which combined with increasing phosphate concentrations results in high CPP levels (Figure 1).

In CKD, serum CPP levels positively correlate with vascular calcification and inflammatory markers.^37–39^ *In vitro* studies have shown that CPPs induce the calcification of VSMCs and the release of inflammatory cytokines from VSMCs and macrophages.^40,41^ However, as described earlier for free inorganic phosphate, the precise molecular mechanism underlying the induction of CPP-mediated cellular effects remains unclear. It is possible that circulating CPPs are taken up by cells and that lysosomes release calcium and phosphate from the CPPs into the cytosol, which are then packaged into calcifying EVs and released as a cellular pass-through system.^14^ However, CPPs might also induce osteoblastic cell differentiation or apoptosis which drives the calcification process. These changes in cell function and survival might not depend on CPP uptake into cells, but on the CPPs' action as extracellular signaling molecules that bind to specific cell surface receptors to induce signaling events.^42,43^ It is also possible that the pathologic actions of circulating CPPs do not directly involve any target cells but are based on the incorporation of the CPP crystals themselves into the altered and calcifying extracellular matrix.^14^ However, a recent tracing study has shown that injected CPPs do not incorporate into preexisting calcified vascular lesions, suggesting that CPPs do not simply supply building material for calcifying structures.^31^

**Figure 2:**
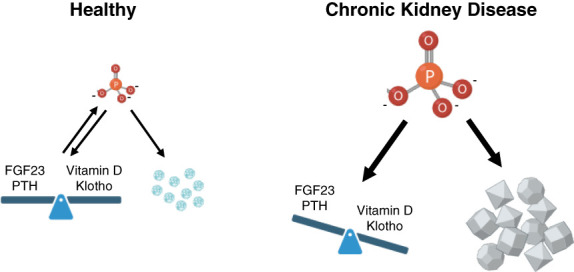
**The potential culprits in CKD-associated hyperphosphatemia.** Pi homeostasis is regulated by the hormones, FGF23, PTH, and vitamin D and by the FGF23 coreceptor klotho. Together, these factors maintain blood levels of inorganic Pi (PO_4_^3−^) in a physiologic range at which Pi cannot crystalize. Instead, Pi and calcium form amorphous particles which can be removed from the circulation. In CKD, reduced renal excretion causes an elevation in inorganic Pi levels in the blood, which results in changes in the endocrine regulators with the goal to maintain Pi homeostasis. With disease progression, the endocrine system eventually fails, leading to highly elevated blood levels of inorganic Pi which together with calcium forms crystals in the blood and in soft tissues. One of the key questions in the field is whether elevated inorganic Pi, the unbalanced endocrine regulators of Pi homeostasis and/or the appearance of calcium-Pi crystals cause tissue damage, such as ectopic calcifications, that occur in CKD. Defining the precise culprit(s) will be important to better understand the underlying pathomechanisms and to identify novel drug targets. FGF23, fibroblast growth factor 23; PTH, parathyroid hormone.

Overall, it remains unclear if the pathologic effects of hyperphosphatemia can be attributed to elevations in phosphate itself or to CPP formation. Furthermore, hyperphosphatemia is accompanied by significant changes in the levels of the endocrine regulators of phosphate homeostasis, *i.e*., elevated FGF23 and PTH and reduced vitamin D and klotho, which themselves could contribute to tissue damage (Figure 2). The complex and multilayered nature of hyperphosphatemia makes it extremely challenging to determine direct versus indirect events and to separate causative actions from bystanders. Moreover, the fact that vascular calcification does not occur homogenously along the vessel wall but in specific areas^44^ suggests that the pathology is not solely driven by elevations in systemic levels of phosphate or CPPs but also includes other factors that might be dysregulated in CKD and that act locally to generate hot-spots for the actions of phosphate and/or CPPs.

## The Challenges in Identifying and Measuring the Pathologic Forms of Phosphate

To date, hyperphosphatemia is defined as a condition where there is too much or excess inorganic phosphate in the blood. Speaking in numbers, hyperphosphatemia has plasma phosphate levels that are higher than 4.5 mg/dl.^45^ Current clinical practice guidelines recommend the use of phosphate binders and dietary phosphate restriction to lower serum phosphate levels toward the normal range in dialysis patients. However, the benefits of these approaches on outcomes, including mortality, and the optimal serum phosphate target have not been tested in larger randomized clinical trials, only in smaller pilot trials.^46,47^ It remains to be determined whether lowering serum phosphate levels toward normal improve outcomes in ESKD.^48^ Two large pragmatic randomized clinical trials in dialysis patients have been designed to tackle this important question. The Higher vs Lower Serum Phosphate Targets in Patients Undergoing Hemodialysis trial (ClinicalTrials.gov NCT04095039) in the United States aimed to investigate the effects of different serum phosphate targets of “Hi” (≥6.5 mg/dl) versus “Lo” (<5.5 mg/dl), achieved by phosphate binder prescriptions and dietary recommendations, on all-cause mortality.^49^ Unfortunately, the trial was terminated early by the External Data Safety Monitoring Board. The ongoing international High or Standard Phosphate Targets in End-Stage Kidney Disease trial (ClinicalTrials.gov NCT03573089) tests the effects of lowering serum phosphate to different target levels through phosphate binders on cardiovascular events and physical health. However, as mentioned before, it remains unclear if elevated inorganic phosphate in the circulation is the true culprit in pathologies driven by phosphate increases. Therefore, negative outcomes in clinical trials measuring serum phosphate concentrations in relation to outcomes might not suggest that elevated phosphate does not contribute to tissue damage and mortality in CKD. In general, as long as it is unclear in which form and at which location elevated phosphate causes pathologic changes, it seems to be impossible to conduct meaningful clinical measurements to study potential causalities between hyperphosphatemia, tissue injuries, and mortality in patients with CKD.

**Figure 3:**
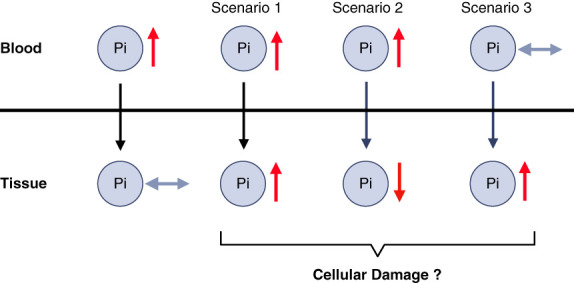
**The relationship between Pi levels in blood versus tissue.** It is important that tissues have the capacity to maintain normal levels of inorganic Pi, even if Pi levels in the blood rise, for example, after food intake. However, very high and prolonged elevations of circulating Pi, as in CKD, seem to affect Pi tissue content in different ways. High blood Pi might result in high tissue Pi (scenario 1), as the case in the calcified vasculature, and most likely in all calcifying tissues, as well as in the lung. High blood Pi can also result in low tissue Pi (scenario 2), as described for skeletal muscle. Finally, some tissues might increase Pi content before a rise in blood Pi (scenario 3), which seems to be the case for the liver. Overall, different tissues seem to show different responses to elevations in blood Pi, which might also depend on the pathologic context. Furthermore, rises in tissue Pi, as the case in scenarios 1 and 3 might not have pathologic effects, at least not initially, but aim to temporarily store Pi or to remove Pi from the system. It is important to note that in scenarios 2 and 3, Pi levels in the blood do not reflect changes in tissue Pi content.

Several scenarios underlying the pathologic actions of hyperphosphatemia on the tissue level seem to be possible (Figure 3). The first and maybe most plausible scenario is that elevations in the levels of inorganic phosphate in the blood and in the extracellular milieu result in increased phosphate uptake by cells and thereby elevations in intracellular levels of phosphate. Cells might have developed protective mechanisms to decrease phosphate import and/or increase phosphate export in the presence of high extracellular phosphate levels to maintain a normal intracellular phosphate milieu. However, such protective mechanisms might be exhausted or fail if phosphate elevations are chronic and persist over time. The second and maybe least logical scenario is that elevations in the levels of inorganic phosphate in the blood and in the extracellular milieu result in decreased intracellular levels of phosphate. The third scenario is that tissue levels of inorganic phosphate increase before a noticeable elevation of phosphate in the blood. It is possible that in this case, phosphate is bound to the extracellular matrix or present inside the cells. Such a mechanism might exist to lower serum phosphate levels, especially when renal excretion is impaired, such as in CKD, with the cost that if the scenario is not reversed, it might result in tissue damage (Figure 1). Cells might have developed mechanisms to store excess phosphate to remove it from the circulation and thereby protect other tissues. Organs differ in phosphate content in relation to their total mass, and therefore, it is likely that different types of cells and tissues also differ in their capacity to take up and store phosphate. It is plausible to assume that in the three described scenarios, either elevations or reductions in intracellular phosphate levels have significant effects on cellular structures and functions, such as ATP synthesis and the activity of ATPases. Clearly, these scenarios are hypothetical and need experimental evaluation. Of note, in scenarios 2 and 3, changes in serum phosphate levels do not correlate with changes in tissue phosphate levels (Figure 3).

Scenario 1 (“more outside—more inside”) is the case for the calcified vasculature in CKD, where the tissues generate a bone-like extracellular matrix.^14^ In general, all tissues that calcify have increased phosphate content based on the presence of calcium-phosphate crystals. However, it is unclear if tissue elevations of phosphate always result in ectopic calcification or if phosphate can be elevated in other forms in the extracellular matrix or within the cell. It is challenging to measure intracellular levels of free phosphate which would be needed to tackle this important question. It has been shown in yeast and in mammalian cells, such as cardiomyocytes, that phosphate can polymerize and form poly-phosphate.^5,50,51^ If poly-phosphate is an intracellular storage form of phosphate or a pathologic form of phosphate that can cause cell damage is unclear.^5^ Furthermore, it has been shown that increased phosphate uptake by mitochondria, which already have high phosphate levels in their matrix, can cause the formation of intraorganelle calcium-phosphate crystals which then cause cell damage from the inside.^52^

Scenario 2 (“more outside—less inside”) might exist for skeletal muscle tissue in CKD. It has been recently shown in a mouse model of CKD, with nephrectomy and the administration of a high-protein diet, that elevations in serum phosphate levels were accompanied by reductions in the phosphate content of skeletal muscle tissue.^53^ These CKD mice also develop sarcopenia, and interestingly, when a certain skeletal muscle-specific protein was genetically deleted, mice were protected from sarcopenia, the muscle content of phosphate increased, and serum levels of phosphate were reduced, while kidney function and urinary phosphate excretion were still reduced. This finding suggests that reduced phosphate uptake capability of skeletal muscle might contribute to serum phosphate elevations in CKD. Furthermore, a recent phosphorus magnetic resonance spectroscopy study in calf muscle of patients with ESKD showed that during a 4-hour hemodialysis session, the skeletal muscle content of free phosphate and ATP significantly reduced, indicating that the dialysis process lowers intracellular phosphate levels in patients with CKD.^54^ Another phosphorus magnetic resonance spectroscopy study in healthy individuals showed that dietary phosphate loading reduces ATP content in skeletal muscle, indicating that high phosphate intake is associated with low muscle phosphate levels.^55^

There are several lines of evidence that scenario 3 (“same outside—more inside”) exists, at least in the context of CKD. For example, acute phosphate elevations in healthy rats by intravenous infusions have shown that after 4 hours, serum phosphate levels are normalized, while only 50% of phosphate are eliminated by the kidney,^56^ suggesting that the other 50% of phosphate have been deposited in tissues. Indeed, this has been shown by phosphate tracing studies in rats with infusions of radioactive phosphate (^33^PO_4_). It was found that tissue can take up phosphate within 30 minutes and that the capacity for acute uptake of phosphate from the circulation differs among tissues.^57^ Kidney, bone, heart, liver, and lung showed highest ^33^PO_4_ levels, while the vasculature and other tissues including skeletal muscle and brain barely took up any phosphate. When infusions were conducted in rats with prior induction of CKD and vascular calcification, most tissues showed elevations in ^33^PO_4_ levels, with highest *de novo* depositions in the vasculature. In a more recent study where ^33^PO_4_ was administered through the diet, ^33^PO_4_ depositions in healthy mice were detected in the liver, as well as kidney and bone, after 6 hours.^58^ Similar to the previous study, when CKD and vascular calcification were induced before the introduction of the ^33^PO_4_ diet, ^33^PO_4_ deposition in the vasculature was massively increased. Interestingly, in this setting, lung tissue showed high increases in phosphate content. Combined, these studies suggest that tissues differ in their capacity for acute phosphate deposition, which is altered if kidney function declines. They also indicate that by accumulating phosphate from the circulation, the calcifying vasculature might mediate nonrenal clearance of serum phosphate, thereby regulating acute phosphate homeostasis.^58^ This buffering mechanism seems to primarily work for amorphous phosphate but not for crystalline CPPs. This finding has been confirmed by another recent tracing study using radioactive and fluorescence-labeled crystallin CPPs injected into rats with CKD and preexisting vascular calcification, where CPPs accumulated in liver, spleen, and lung, but not in the calcified vasculature.^31^ In healthy animals, the liver seems to take up significant amounts of phosphate after acute elevation.^58^ Furthermore, elevating dietary phosphate content in healthy mice for 12 weeks increased serum phosphate levels which positively correlated with increases in hepatic phosphate levels.^18^ Interestingly, the rise in phosphate content of the liver occurred before the elevation of serum phosphate levels. Although hepatic uptake removes CPPs from the circulation,^31^ as mentioned earlier, the faith of inorganic phosphate that is taken up by the liver is unclear. It could be speculated that the liver provides a reservoir for storing excess phosphate, which can be released back into the circulation when serum phosphate levels drop.

**Figure 4:**
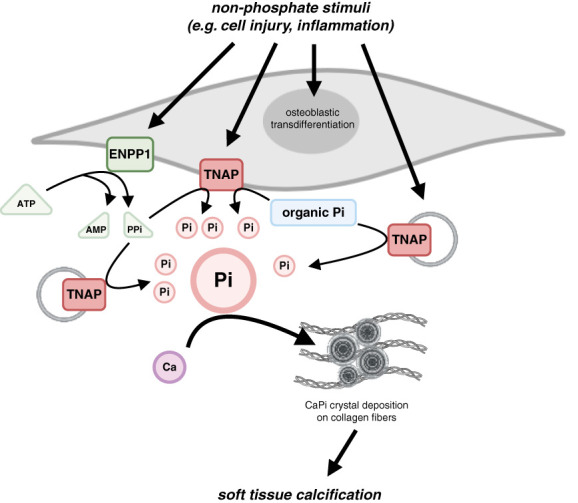
**The regulation of local Pi levels.** Cells seem to be able to regulate the concentration of inorganic Pi in their extracellular environment by expressing enzymes on their surface that can generate Pi. This includes ENPP1 which generates PPi and AMP from ATP, and TNAP, which then hydrolyzes PPi to generate free Pi. TNAP can remove Pi from various substrates, including several forms of organic Pi. Cells can also release EV that contain TNAP which generates free Pi within the extracellular matrix. The relevance of this mechanism under physiologic conditions is not understood. If uncontrolled and accompanied by the activation of osteogenic gene programs, elevated Pi and calcium form crystals that are deposited on collagen fibers and cause tissue calcifications. Importantly, this cellular process to increase local Pi content is not initiated by elevations in blood Pi levels, but by other pathologic stimuli. Ca, calcium; CaPi, calcium-phosphate; ENPP1, ectonucleotide pyrophosphatase/phosphodiesterase 1; EV, extracellular vesicle; Pi, phosphate; TNAP, tissue-nonspecific alkaline phosphatase.

Scenario 3 (“same outside—more inside”) is also supported by findings that cells can regulate the content of free inorganic phosphate in their extracellular environment which seems to be independent of systemic phosphate levels (Figure 4). Ectonucleotide pyrophosphatase/phosphodiesterase 1 (ENPP1) is a membrane-bound enzyme that hydrolyzes extracellular ATP into AMP and PPi, thereby acting as the primary source of extracellular PPi in the body.^59^ Tissue-nonspecific alkaline phosphatase (TNAP) that is also on the cell surface hydrolyzes PPi and generates free phosphate.^60^ TNAP also removes phosphate groups from organic phosphate, such as ethyl phosphate or glycerona phosphate, thereby serving as a major generator of inorganic phosphate.^61^ By controlling local levels of PPi versus phosphate, this system serves as an important regulator of mineralization in osteoblasts and osteocytes and of bone formation.^62^ ENPP1 and TNAP also seem to be activated in other cell types outside of the bone, at least under pathologic conditions. For example, calcifying VSMCs express TNAP on the cell surface as well as on EVs, which increases local phosphate levels and promotes the formation of calcium-phosphate crystals in the matrix.^14^ This process is independent of serum phosphate levels and seems to be induced by other CKD-associated pathologies, such as inflammation. However, since animal models of CKD *per se* do not usually develop vascular calcifications, and require an additional elevation of dietary phosphate content to do so,^63^ the generation of local phosphate pools within the vasculature might not be sufficient to induce vascular calcification. A similar mechanism of local phosphate elevations might also be activated in the heart in response to injury.^64^ A study in mice has shown that cardiac fibroblasts in the injured myocardium start to express ENPP1 and TNAP and eventually calcify, which drives local calcifications, also called dystrophic calcification.^65^ Interestingly, calcifying fibroblasts maintain their molecular makeup and can induce calcifications when transferred into healthy mice. Although the significance of this local calcification mechanism outside of bone tissue needs to be studied in more detail and in various cell types and contexts, it provides insights into the existence of sophisticated local mechanisms that cells have developed to regulate the phosphate content in their surroundings. The biologic relevance of local phosphate changes needs to be determined, but it can be assumed that when out of control, they can contribute to pathologies, such as the formation of calcium-phosphate crystals.

**Figure 5:**
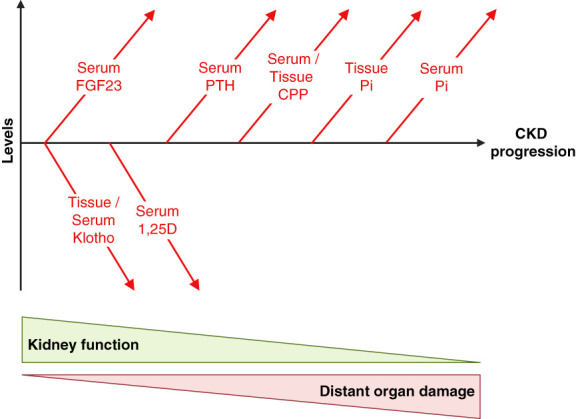
**The changes in Pi metabolism in the course of CKD progression.** With declining kidney function, the endocrine regulators of Pi metabolism undergo significant changes in their serum levels to compensate for the reduced capacity of renal Pi excretion with the goal to keep systemic Pi concentrations in a normal range. The levels of FGF23 and klotho seem to be altered first, followed by changes in active vitamin D (1,25D) and PTH. The serum levels of inorganic Pi only increase in very late stages of CKD, raising the question whether Pi can contribute to distant organ damage that is initiated early in CKD. While the described alterations in Pi metabolism are well established, more recent research has shown that Pi-containing CPP appear early in the blood. Furthermore, CPPs and inorganic Pi might accumulate in tissues before a rise in serum Pi levels. It is possible that these earlier elevations in CPPs and in tissue Pi content contribute to CKD-associated tissue damage.

Serum levels of inorganic phosphate increase very late during CKD progression^66^ (Figure 5), raising the question if hyperphosphatemia can actually be a cause of CKD-associated injury, especially in pathologies that appear early during disease progression. However, it is possible that as described for scenario 3 (Figure 3), tissues accumulate phosphate in early CKD stages before serum phosphate levels rise and that these local phosphate accumulations contribute to tissue injury. In general, since almost all of the body's phosphate is found in bone and in soft tissues and only <1% in extracellular fluids including the blood,^1,6^ it is likely that serum measurements of inorganic phosphate do not reflect overall phosphate levels in health or disease. Furthermore, CPPs have been detected in the blood of patients with CKD, even in early disease stages when serum phosphate levels are not significantly elevated^37,39^ (Figure 5). It is possible that in CKD, removal of CPPs by the liver is impaired or oversaturated and that elevated circulating CPPs cause tissue damage, which occurs in the presence of normal serum phosphate levels (Figure 1). It has been shown in rats receiving a high-phosphate diet for 10 weeks that serum levels of CPPs but not of phosphate increased,^67^ suggesting that also in the healthy state, significant elevations in CPP levels can precede the detectable increase in inorganic phosphate concentrations in the blood.

Clearly, measuring serum CPP levels as well as levels of inorganic phosphate in tissues would help to determine the contribution of phosphate to CKD-associated pathologies and mortality. However, although inorganic phosphate can be easily measured through colorimetric assays, which is the clinical standard, intracellular phosphate concentrations are difficult to precisely quantify because most of the phosphate is bound and levels may vary substantially between cell types.^68,69^ Furthermore, tissue studies would require biopsies, and since different tissues might differ in their changes in phosphate content, measuring one type of tissue might not be sufficient. Measuring CPPs in the blood can be challenging, as CPPs cannot be detected by conventional phosphate assays but require the detection of fetuin-A^37,39^ or the use of bisphosphonates which specifically bind CPP crystals.^67,70^ CPPs can be also indirectly detected by the serum T50 test that captures the transformation or ripening time of CPPs.^71^ The test measures the rate by which primary, amorphous CPPs are transformed into secondary, crystalline CPPs in a blood sample. Lower serum T50 values indicate shorter conversion time and higher propensity for pathophysiologic changes and have been associated with vascular calcification, cardiovascular events, and mortality in patients with CKD.^71–75^

## Conclusions

There is strong experimental and clinical evidence that hyperphosphatemia is the main cause of vascular calcification in CKD.^14,15^ However, it remains unclear if inorganic phosphate acts on its own, by forming crystals with calcium, or by altering the endocrine system that regulates mineral metabolism. Without a clear picture of the true culprit, it is not surprising that the direct targets and pathomechanisms of hyperphosphatemia are not well described. Furthermore, since we are missing a deeper mechanistic understanding of hyperphosphatemia-driven pathologies, it is also not surprising that current phosphate-lowering agents seem to lack efficacy. Clinical CKD trials using phosphate binders showed mixed results in reducing serum phosphate levels and in improving cardiovascular outcomes and survival,^45,76^ and therefore, it remains unclear if phosphate binders, as currently deployed, improve outcomes in ESKD. Maybe it is time to take a step back and to look at the bigger picture for the potential actions of inorganic phosphate, which includes the systemic versus local actions of phosphate, the actions of phosphate versus phosphate-containing particles, and the mechanisms that protect from calcium-phosphate crystal formation and that might fail in CKD. One should not assume that hyperphosphatemia is a homogenous event that effects all tissues and all cells within a tissue to a similar degree. Furthermore, one should not assume that all hyperphosphatemia-associated pathologies are driven by elevations of inorganic phosphate in the circulation. It will be important to design tools to measure phosphate in tissues and to distinguish between inorganic phosphate itself versus phosphate-containing particles. Research to identify the pathologic forms and actions of phosphate will not only help to develop novel drugs for patients with CKD but should also have wider effects. It should help to determine if such mechanisms also affect other cell types than VSMCs and drive other pathologies than vascular calcification. Furthermore, it will help to determine if phosphate in other contexts than CKD can cause tissue damage, such as during the normal aging process or with high dietary phosphate load. Since CKD patient numbers are extremely high, the phosphate-rich Western diet is popular and on the rise, and efforts to understand aging and increase lifespan are growing, it is worth looking into phosphate as a potential culprit that can harm various types of cells and tissues.
